# Task-Irrelevant Semantic Properties of Objects Impinge on Sensory Representations within the Early Visual Cortex

**DOI:** 10.1093/texcom/tgab049

**Published:** 2021-08-10

**Authors:** Joseph C Nah, George L Malcolm, Sarah Shomstein

**Affiliations:** Center for Mind and Brain, University of California at Davis, Davis, CA 95618, USA; School of Psychology, University of East Anglia, Norwich NR4 7TJ, UK; Department of Psychological and Brain Sciences, The George Washington University, Washington, DC 20052, USA

**Keywords:** early visual cortex, fMRI, parietal cortex, semantic influence, visual attention

## Abstract

Objects can be described in terms of low-level (e.g., boundaries) and high-level properties (e.g., object semantics). While recent behavioral findings suggest that the influence of semantic relatedness between objects on attentional allocation can be independent of task-relevance, the underlying neural substrate of semantic influences on attention remains ill-defined. Here, we employ behavioral and functional magnetic resonance imaging measures to uncover the mechanism by which semantic information increases visual processing efficiency. We demonstrate that the strength of the semantic relatedness signal decoded from the left inferior frontal gyrus: 1) influences attention, producing behavioral semantic benefits; 2) biases spatial attention maps in the intraparietal sulcus, subsequently modulating early visual cortex activity; and 3) directly predicts the magnitude of behavioral semantic benefit. Altogether, these results identify a specific mechanism driving task-independent semantic influences on attention.

## Introduction

The visual system extracts high-level semantic information from complex scenes early in the information processing stream (Potter 1976; Thorpe et al. 1996; Potter et al. 2014) and attentional allocation is then guided by more detailed semantic signal such as object and scene layout (Loftus and Mackworth 1978; Brockmole et al. 2006; Castelhano and Henderson 2007), the relationship between a scene and object (Malcolm and Henderson 2010; Võ and Henderson 2010; Spotorno et al. 2014), and the co-occurrence and spatial dependency between objects in scenes (Bar 2004; Oliva and Torralba 2007; Mack and Eckstein 2011). Additionally, behavioral findings indicate that semantic relatedness of objects guides attention when task-relevant (Moores et al. 2003; Belke et al. 2008; Castelhano and Heaven 2010; de Groot et al. 2016). Taken together knowledge-based information plays a crucial role in attentional guidance.

Despite repeated demonstrations that task-relevant information guides selective attention, most of the information in the environment is task-irrelevant. For instance, when stopped at the intersection, the color of the traffic light, vehicles, and pedestrians are immediately relevant. However, these task-relevant details of the scene explain only a fraction of the environment. Buildings, benches, mailboxes, etc., are all processed perceptually, while irrelevant to the task of driving. Whether these task-irrelevant aspects exert similar influence on attention is unclear. If relevant and irrelevant semantic information guides attention, it suggests an automatic nature of such influence. Thus, once objects are processed, we are aware of and can be influenced by their high-level properties whether that information is directly relevant (Shomstein et al. 2019). Recent evidence posits an influence of high-level properties of objects on attentional allocation when task-irrelevant. For instance, semantic relationships between objects bias spatial attentional allocation (Malcolm et al. 2016), out-of-place objects (e.g., toothbrush on desk) are fixated for longer than control items (Cornelissen and Võ 2017), and meaningful regions within a scene are fixated more than salient regions (Peacock et al. 2018). Combined with the speed at which high-level information is processed (Potter and Levy 1969; Potter 1976; Thorpe et al. 1996; Potter et al. 2014), these results demonstrate that semantic information has a continuous, and likely automatic, influence on attention (Shomstein et al. 2019).

In order to posit that object semantics influence attention, such information has to be computed and be readily available. Recent neuropsychological and neuroimaging literature provides evidence that semantic information is derived by a broadly distributed neural network, lateralized toward the left hemisphere (Binder et al. 2009). Specifically, the left inferior frontal gyrus (IFG) has been demonstrated to be crucial in the control of semantic information, including the retrieval and evaluation of meaning (Gabrieli et al. 1996; Fiez 1997; Thompson-Schill et al. 1997; Wagner et al. 1997; Wagner et al. 2001) and as a key region that computes semantic similarity (Carota et al. 2017). Additionally, the left posterior temporal lobe is implicated in storing object representations (Martin 2007) with some claiming that the temporal lobe is the “hub” for semantic representations (Patterson et al. 2007). Additionally, several recent studies point to the pervasiveness of task-relevant semantic information within the perceptual system (Thompson-Schill et al. 1997; Bar and Aminoff 2003; Brady and Oliva 2008; Binder et al. 2009; Lupyan et al. 2010; Greene and Fei-Fei 2014; Livne and Bar 2016) with direct evidence of semantic representations decoded throughout the ventral visual cortex, from the occipital to temporal pole (Çukur et al. 2013).

**Figure 1:**
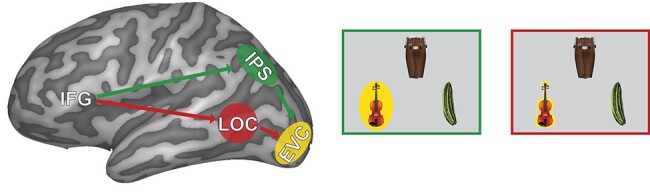
Predicted pathway of semantic influence. If semantic information biases spatial attention (green path + green bordered box), the spatial location occupied by the SR object is preferably processed. If semantic information influences object representations (red path + red bordered box), the SR object itself is preferably processed.

While prior investigations identified brain regions sensitive to semantics and demonstrated that semantics influences behavior, these two types of observations remained largely independent. Consequently, the precise mechanism of how this information influences attentional distribution remains ill-defined. In service of this question, we directly tested whether attentional benefit from task-irrelevant semantic information modulates sensory representations of objects in the early visual cortex (EVC) through facilitation of spatial representations in spatially selective intraparietal sulcus (IPS) (Bisley and Goldberg 2010; Shomstein and Gottlieb 2016; Todd and Manaligod 2017), or object representations derived in the object-selective lateral occipital complex (LOC), or possibly both (Fig. 1). Thus, the present report: 1) establishes that semantic information influences behavior automatically, independent of task-relevance, and 2) elucidates the neural mechanism through which task-irrelevant semantic information influences attentional selection.

## Materials and Methods

### Participants

Sample size was determined by adopting similar sample sizes used in previous neuroimaging studies (Bar and Aminoff 2003; Walther et al. 2009; Livne and Bar 2016) combined with multiple trials (many observations, multiple scanning sessions) per experimental run for each participant. Twelve participants (four females; mean age 25.1, range 20–33) with normal or corrected-to-normal vision, and no history of neurological problems, participated in Experiment 1. One participant was removed from data analysis (chance level accuracy). Fourteen participants (10 females; mean age: 23.7, range: 19–29) with normal or corrected-to-normal vision, and no history of neurological problems, participated in Experiment 2. One participant was removed from data analysis due to excessive head motion within the scanner. Four participants took part in both Experiments 1 and 2. All participants gave informed consent and the study was approved by the Institutional Review Board of The George Washington and Georgetown Universities.

### Statistical Analysis

All statistical analyses were performed in SPSS or MATLAB using custom code. Behavioral data within the manuscript are presented as the mean or accuracy ±1 standard error of the mean (SEM) corrected for within-subjects variance (Cousineau (2005)), unless otherwise noted. Behavioral data obtained from the scanner were analyzed using a paired sample *t*-test. Unless otherwise noted, all functional magnetic resonance imaging (fMRI) analyses were corrected for multiple comparisons by using whole-brain False Discovery Rate of *q* < 0.05.

### Experimental Procedure

#### Experiment 1

**Figure 2:**
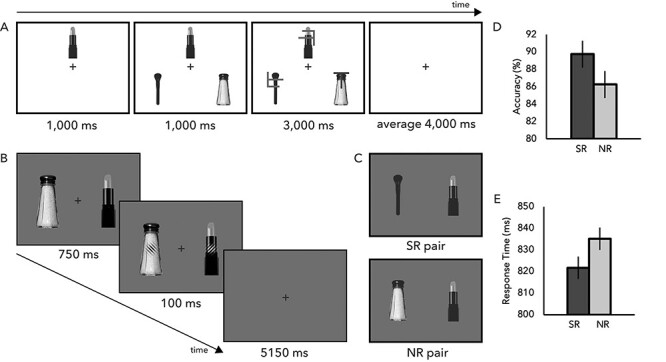
Experiment paradigms and behavioral results. (*A*) Experiment 1—participants were presented with a reference object presented above the fixation cross. Next, two objects appeared in periphery below the midline creating an object triad. One of the peripheral objects was SR to the central object, while the other was not (NR). After a 1-s delay, a target letter (T or L) along with two distractor characters (T/L hybrids) appeared superimposed on top of the objects. Participants then performed target discrimination task. Semantic relatedness was not predictive of target location, thus task-irrelevant. (*B*) Experiment 2—participants were presented with two objects appearing on either side of fixation. The objects were either SR to one another, or not. A rotated Gabor patch was overlaid on top of either object, with participants tasked to report whether the two Gabor patches matched in orientation. (*C*) Example of SR and NR pairs in Experiment 2. (*D*) Experiment 1 behavioral results—participants were marginally more accurate at identifying targets that appeared on top of an SR object. (*E*) Experiment 2 behavioral results—participants were more accurate and significantly faster at performing the task when the objects were SR (error bars represent ±1SE).

Each trial began with a reference object presented just above the central fixation cross (Fig. 2*A*). After 1000 ms, two objects appeared in each periphery below the midline creating a triangle, or “triad.” Critically, one of the peripheral objects was always semantically related (SR; objects that frequently co-occur in the real-world or functionally go together) to the central object, while the other was not related (NR) (e.g., if the central object was a lamp, peripheral objects were a light bulb and an envelope). Participants were instructed to maintain fixation and to prioritize speed of response (focusing on accuracy analysis). No other eye movement controls were included in Experiment 1 (but later controlled for in Experiment 2). After the triads remained on screen for 1000 ms, a target letter (T or L) along with two distractor characters (T/L hybrids) were superimposed on top of the objects (one on each object). The long object exposure was intended to allow sufficient time to fully process the object information as well as any semantic association between them. The aim was to test whether semantic relatedness of fully recognized yet task-irrelevant objects has consequences for attentional allocation. Participants then performed a target discrimination task while maintaining fixation on the central cross. Most importantly, the target appeared on the three objects with equal probability, rendering the semantic information between objects irrelevant to the task at hand (i.e., actively attending to the semantically related [SR] object would not be beneficial in performing the task). Participants had 3000 ms to respond, after which the triad was removed from screen. The intertrial stimulus interval (ISI) was jittered between 2000 and 6000 ms in 250-ms steps for an average of 4000 ms to allow for BOLD deconvolution. Participants completed a total of seven experimental blocks (runs) intermixed between functional localizer scans.

#### Experiment 2

Each trial began with two objects appearing on either side of a center fixation for 750 ms. A vertical Gabor patch rotated 45° horizontally in either the left or right direction, was then overlaid on top of each object for 100 ms followed by an ISI of 5150 ms (Fig. 2*B*). Participants were instructed to report whether the two Gabor patches were identical in orientation. The two objects on the screen were either SR or NR to one another (e.g., a makeup brush and lipstick or a makeup brush and salt shaker; Fig. 2*C*). Participants performed a total of 48 trials per run, for a total of 8 runs, with an equal number of trials for each condition. Participants were instructed to respond as fast as possible without sacrificing accuracy (focusing analyses on response times [RTs]).

### fMRI Acquisition Parameters

MRI scanning was conducted at the Center for Functional and Molecular Imaging at the Georgetown University Medical Center on a Siemens 3-Tesla scanner equipped with a 12-channel head coil. All functional data during the experimental as well as localizer runs were acquired in 38 transverse slices covering occipital and parietal cortices (TR: 2000 ms, TE: 30 ms, matrix size: 64 × 64, FoV: 192 mm, acquisition voxel size: 3 × 3 × 3 mm). High-resolution anatomical images (1 mm^3^) were also acquired for each participant using MPRAGE *T*_1_-weighted sequence (TR: 1900 ms, TE: 2.5 ms, matrix size: 256 × 256, FoV: 250 mm). In both experiments, each participant completed a single 1.5 hour-long session which included either 7 (Experiment 1) or 8 (Experiment 2) experimental runs and functional localizers (5 runs). Custom E-Prime (Sharpsburg, PA) scripts were used to generate the main experimental task of Experiment 1 and custom Python code using the PsychoPy library (Peirce 2007, 2009) were used to generate Experiment 2 and for the localizer scans for both experiments. The display was then back projected onto a screen mounted at the rear end of the scanner, which participants viewed via a mirror attached to the head coil (distance from screen to mirror: 80 cm, average distance from participant’s face to mirror: 10 cm).

### fMRI Preprocessing and Analysis

All data were preprocessed using BrainVoyager QX (version 2.8.0, Brain Innovation) along with custom MATLAB and Python scripts. All functional data were slice time corrected, motion corrected, and temporally high-pass filtered using a general linear model (GLM) containing a Fourier basis set (2 sines and cosines) to remove low- and high-frequency noise in the functional time series. All EPI and anatomical images were then normalized into Talairach space (Talairach and Tournoux 1988) and interpolated into 1-mm isotropic voxels. Localizer data as well as data used for univariate analysis were spatially smoothed with a 3-mm FWHM kernel. A segmented white matter mask generated from FreeSurfer (recon-all function) was imported into BrainVoyager to segment white matter from gray matter. Afterward, the cortical surface for each hemisphere was inflated and all analysis was conducted on the inflated surface.

#### Experiment 1—Univariate Analysis

Within each region of interest (ROI), the average Blood Oxygenation Level-Dependent (BOLD) response magnitude across was calculated for each condition (SR, NR). Triad-related activity (1TR), time-locked to the onset of the completion of object triads (i.e., once all three objects were presented on screen), was deconvolved to estimate the hemodynamic response function for each event type. The BOLD response was estimated at the onset of the triad and at each of the next 12 time points, 0–24 s after stimulus. The BOLD peak response for each condition was calculated by averaging coefficients at time point 6.

Preliminary analysis was conducted separately for each hemisphere, consistent with the experimental design. For instance, if an SR object appeared on the left side of fixation, an NR object would appear simultaneously on the right. Thus, the neural activity for SR was examined within the right hemisphere ROI, while the neural activity for NR was examined within the left hemisphere. Semantic relationship beta coefficients were extracted separately from each ROI by using a GLM and a design matrix that modeled the response.

#### Experiment 2—Multivariate Analysis

Multivariate pattern analysis was conducted to assess changes in the pattern of activity of each EVC ROI modulated by task-irrelevant semantic information. Custom MATLAB code using LIBSVM (Chang and Lin 2011) as well as BrainVoyager’s multivariate pattern analysis (MVPA) module was utilized to conduct these analyses. For each individual trial in each condition, *t*-values (Misaki et al. 2010) were extracted by fitting a canonical two-gamma HRF in a standard GLM regression analysis and z-score normalized to control for differences in baseline activity (Kravitz et al. 2011). The resulting *t*-values were then used as feature vectors for that trial or block and used to train a linear support vector machine (SVM). To assess classification performance, the leave-one-run-out cross-validation was utilized. On each iteration, 7 experimental runs were used to train the classifier and the remaining run was used to test the classifier’s performance. The classification performance was then averaged across all cross-validation loops to obtain average classification accuracy for each ROI and condition. This accuracy was then compared with chance performance (25%) as well as between SR and NR conditions.

### Functional Localizers and Retinotopic Mapping

For each participant, separate sets of functional localizers were conducted to define ROIs: 1) meridian localizer to delineate borders between EVC dorsal regions V1, V2, and V3 (and an additional stimuli position localizer for Experiment 1 to identify patches of cortex responsive to the object locations); 2) LOC localizer to identify object-selective regions of cortex; and 3) IPS localizer to define spatially selective regions of cortex (see Supplementary Tables 1 and 2 in supplementary materials for ROI coordinates and size).

1. Meridian and stimuli position localizer: Retinotopic areas (V1, V2, and V3) were defined using flashing checkerboard stimuli presented in a bowtie shape that flipped between blocks of vertical and horizontal meridians. Each half of the bowtie was a 16° wedge flickering at 5 Hz. This allowed delineation of borders between dorsal and ventral retinotopic regions of the visual cortex. Concurrently, participants fixated on a central black fixation square that randomly dimmed to gray for variable durations. Participants were informed to hold down a button every time the black fixation square dimmed to gray and release it when black. Each meridian was presented 8 times for 12 s starting with the horizontal meridian and was bookended by 8-s fixations for a total scan time of 208 s. An additional stimuli position localizer was conducted to define the spatial locations (bottom left and bottom right) occupied by the objects in the experimental task and increase the selectivity of voxels. Participants maintained fixation at the center of the display and pressed a button whenever a small gray dot appeared in the middle of the fixation cross. Concurrently, flashing checkerboard stimuli (2.7° × 2.1°) flickering at 5 Hz was presented in one of the three possible stimuli location (above fixation, lower-left, lower-right) for 12 s. The initial fixation duration was 10 s, and each location block lasted 12 s, with an 8-s interblock fixation and a final fixation of 6 s for a total scan time of 256 s. The spatial location of the stimuli was then functionally defined with a lower-left > lower-right contrast for the left object and lower-right > lower-left for right object. Final EVC ROIs were determined by restricting the voxels to the overlapping activations from both the meridian and position localizer. ROIs were drawn for each participant for each hemisphere.

2. LOC localizer: All stimuli were presented at the center of the screen, with a fixation overlaid on top of all objects. A stream of 16 stimuli was presented one at a time for 500 ms with an interstimulus interval of 500 ms for a total of 8 s per block. Participants were informed to maintain fixation and perform a one-back task, identifying repeated images via a button press.

3. IPS localizer: Spatially selective regions (IPS: IPS0, IPS1, IPS2) were defined using a random dot movement change detection task. Two circles (3°) in opposite corners (upper left and lower right, lower left and upper right) were displayed simultaneously with the circle pairs alternating each block. Within the circles were dynamic random dot stimuli (RDS) moving horizontally. Concurrently, participants fixate on a central cross which indicated a direction either up or down with a change in color in one of the arms (e.g., the upper half of the vertical line would change from gray to green to indicate the direction, up). After a random interval between 500 and 1500 ms, the RDS in one of the two circles changed directions to either up or down. Participants were instructed to indicate with a button press whether the change in direction matched the direction displayed on the fixation cross. Contrasting the two conditions approximately portrays the full array of visual space. IPS0, IPS1, and IPS2 were demarcated by visually inspecting reversals in activation as well as anatomical guidelines (Sheremata 2019).

### Stimuli and Low-Level Control Analysis

#### Experiment 1

Stimuli were directly adopted from a previous study, which were controlled for low-level differences (Malcolm et al. 2016). To quantitatively measure the semantic similarity between objects, all object relationships were analyzed through the linguistics-based computational method known as latent semantic analysis (LSA) (Landauer and Dumais 1997). LSA extracts the contextual meaning of words by statistical computations applied to a large body of text and assumes that words with similar meanings occur more frequently together (Landauer et al. 1998). A high dimensional semantic matrix is then constructed from the large body of text and a technique known as singular value decomposition is applied to simplify the matrix. Afterward, the semantic similarity between two terms is calculated as the cosine value between the two corresponding terms in the semantic matrix. A cosine value closer to 1 indicates that two terms are highly SR with one another, while a value closer to 0 indicates that the two terms are NR. For the current study, LSA was applied to the stimuli combinations represented in text form to compute the semantic similarity using the LSA@CU text/word LSA tool developed by the University of Colorado at Boulder. The semantic similarity between objects in the SR condition (0.32) was significantly greater than that between objects in the NR condition (0.05), *t*(19) = 4.37, *P* < 0.001, Cohen’s *d* = 0.98. Additionally, a separate set of participants (*n* = 28) participated in a semantic rating survey. Participants were presented with images of all possible object pairs from both SR and NR conditions and were asked to rate how related the two objects are on a scale of 1 (not related) to 6 (very related). Paired samples *t*-test revealed that the semantic relatedness between objects in the SR condition (*M* = 5.28) was significantly greater than that between objects in the NR condition (*M* = 1.48), *t*(19) = 30.98, *P* < 0.001, Cohen’s *d* = 6.93.

#### Experiment 2

To rule out the possibility that any semantic influence was confounded by low-level factors (i.e., color and size), the feature space between the main object and their respective SR objects was compared with the differences between the main object and their respective NR objects (Malcolm et al. 2016). First, each object was converted into LAB color space, breaking down pixel information into luminance, green–red, and blue–yellow color channels. For each channel of each object, histograms were created and bin-to-bin comparisons were conducted by comparing the differences between the main object and its respective SR and NR object. Thus, low-level information in a specific location of the main object was directly compared with the corresponding location of the SR or NR object. Smaller differences between objects represented greater similarity in that specific channel. Thus, if the semantic influence was confounded by low-level similarities, significantly smaller differences in one or more channels between the main and SR objects than between the main and NR objects should be observed. However, paired *t*-tests for all three channels did not reveal any significant differences (*t*s < 1), suggesting that the observed semantic effect was not a byproduct of low-level similarities. Lastly, each object image was converted into the number of pixels and compared across conditions to test whether there was a significant difference in the retinal size of the objects. Again, a paired *t*-test did not reveal any significant difference (*t* < 1) between the two conditions, suggesting that the objects used in both conditions were of similar sizes.

Semantic similarity between objects was measured using both LSA and the semantic rating survey. The LSA analysis revealed that the semantic similarity between objects in the SR condition (0.52) was greater than that between objects in the NR condition (0.10), *t*(3) = 6.42, *P* = 0.008, Cohen’s *d* = 3.21. The semantic rating survey also demonstrated that the semantic similarity between objects in the SR condition (*M* = 5.17) was also significantly greater than that between objects in the NR condition (*M* = 1.41), *t*(3) = 20.42, *P* < 0.001, Cohen’s *d* = 10.21.

## Results

### Experiment 1: Task-Irrelevant Semantic Relationships Modulate Spatial Attention

The influence of task-irrelevant semantic knowledge on selective attention was examined through a combination of behavioral and fMRI methods. A key feature of this design is that semantic relationship did not predict the location of the target letter (Fig. 2*A*). Considering the difficulty of the task based on the distance of objects from fixation, participants were instructed to maintain fixation and to respond without sacrificing accuracy.

Behavioral data acquired during the neuroimaging scan provides evidence for task-irrelevant semantic guidance of attention independent of task-relevance. Examination of accuracy rates revealed that participants were marginally more accurate at identifying the target when it appeared on an SR object than when it appeared on an NR object, *t*(10) = 2.21, *P* = 0.052, Cohen’s *d* = 0.665 (Fig. 2*D*). These results are consistent with previous findings reported by our lab (Malcolm et al. 2016) as well as others (Cornelissen and Võ 2017; Peacock et al. 2018), demonstrating that the task-irrelevant semantic relationship between objects influences attentional allocation. Examination of the RT data revealed no significant difference between the two conditions *t* < 1, *P* > 0.4, providing evidence against speed accuracy tradeoffs. The presence of a behavioral effect is an important starting point for the subsequent neuroimaging analyses.

### Semantic Relationship Modulates IPS and EVC Activity

Having demonstrated the influence of task-irrelevant semantic relationships on attentional allocation in this, adopted for fMRI, paradigm, we next examined the underlying neural mechanism. We hypothesized that semantic information either: 1) influences the spatial representations in the IPS, or 2) object representations in the LOC. To distinguish between these two mechanisms, we first tested whether semantic information modulates BOLD responses in the EVC, IPS, and LOC using univariate methods. If task-irrelevant semantic information facilitates neural responses in accordance with the behavioral profile reported above, we expect a greater target-evoked BOLD response for the SR than NR object.

**Figure 3:**
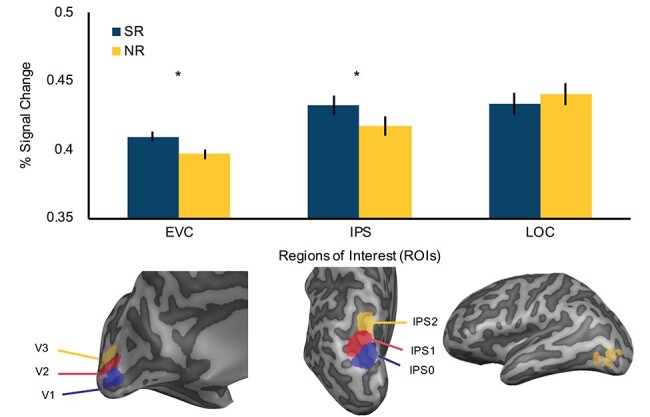
Experiment 1 fMRI results. Semantic relationships significantly modulate activity in EVC and IPS, but not LOC. This suggests that high-level semantic relationships facilitate activity in lower processing regions via an attentional, rather than object recognition mechanism. (^*^P < 0.05, and error bars represent ±1SE).

A three-way repeated measures ANOVA was conducted with semantic relationship (SR, NR), hemisphere (left, right), and externally functionally defined ROIs (V1, V2, V3; see Methods) as within-subject factors to test whether task-irrelevant semantic information influences cortical activity in the early sensory regions. Given the absence of main effects or interactions involving hemisphere and ROI (*F*s < 1), data were collapsed over these factors. Consistent with effects of semantic bias, there was a significant main effect of semantic relationship on BOLD signal, with greater BOLD responses for SR than NR objects (*F*(1,10) = 16.44, *P* = 0.002, *η*^2^*_p_* = 0.62; Fig. 3, EVC). The robust effect of semantic information on cortical activity in the EVC suggests that task-irrelevant high-level information spatially biases attention toward the SR object, resulting in signal enhancement in the early sensory regions, thereby increasing efficiency of visual processing. These results corroborate the behavioral findings reported above and provide strong support for an automatic influence of semantic processing on efficiency of perception.

Next, to determine whether the modulation of EVC activity is accompanied by spatial attentional modulation or direct enhancement of object representations, we investigated whether semantic information modulates activity in the spatially selective IPS and the object-selective LOC. First, a three-way repeated measures ANOVA with semantic relationships (SR, NR), hemisphere (left, right), and externally functionally defined IPS ROIs (IPS0, IPS1, IPS2; see Methods) was conducted. Again, no main effects or interactions involving hemisphere or ROIs reached significance (*F*s < 1), thus data were collapsed over these factors. Similar to the results of the EVC, there was a significant main effect of semantic relationships with greater peak BOLD responses for SR than NR objects, (*F*(1,10) = 5.55, *P* = 0.04, *η*^2^*_p_* = 0.36; Fig. 3, IPS).

Activity in the externally functionally derived object-selective LOC was examined to determine whether object representations for SR objects were directly enhanced. A repeated measures ANOVA with semantic relationships (SR, NR) and hemisphere (left, right) as factors revealed no significant interactions or main effects (*F*s < 1; Fig. 3, LOC), offering no evidence that semantic information influences object representations in LOC. To test whether this difference between semantic relationships in IPS is significantly different from that in LOC, a repeated measures two-way ANOVA with ROIs (LOC, IPS), hemisphere (left, right), and semantic relationships (SR, NR) was conducted, revealing a significant interaction between semantic relationships and ROIs, *F*(1,10) = 5.47, *P* = 0.04, *η*^2^*_p_* = 0.35. Simple main effects analysis indicated that the interaction was driven by the significant difference between SR and NR conditions in the IPS (*F*(1,10) = 5.48, *P* = 0.04, *η*^2^*_p_* = 0.35). No other interaction or main effects were significant (*F*s < 2.3, *P*s > 0.16). Combined, these results suggest that the task-irrelevant semantic relationship between objects modulates neural activity in the EVC through attentional facilitation via the parietal cortex (IPS) rather than directly strengthening object representations in LOC.

Results of Experiment 1 show that semantic relationships influence spatial attentional maps in the parietal cortex and the relative salience of the space in which a semantically related but task-irrelevant object is located. In contrast, no modulation was observed in the ventral processing region LOC, suggesting that semantic information influences activity in the IPS without directly influencing object representations. This finding aligns with previous research demonstrating that LOC is sensitive to the overall physical features of an object (e.g., shape), rather than higher level semantic information (Grill-Spector et al. 1999; Chouinard et al. 2008; Kim et al. 2009). These data provide the first neuroimaging evidence of the pervasive nature of semantic information on attentional allocation, with its influence observed to be independent of task-relevance. Thus, the attentional priority map within the parietal cortex is composed of a multidimensional priority map that incorporates multiple factors in the natural environment to bias attentional allocation (Shomstein and Gottlieb 2016; Shomstein et al. 2019), such as semantic relatedness or category membership (Freedman and Assad 2006).

### Experiment 2: Semantic Relationships Facilitate Attentional Allocation

Experiment 1 demonstrated that semantic information modulates a spatial attentional map within the parietal cortex, which in turn facilitates responses in the early sensory regions. Experiment 2 investigated the consequence of this spatial bias and the neural representation of task-irrelevant semantic relationships. The goals of Experiment 2 were to: 1) demonstrate that the effects of semantic influence are not specific to a particular paradigm by internal replication using a different paradigm and a different neuroimaging analysis method, 2) investigate the consequence of attentional bias on object representations in EVC, and 3) examine whether regions sensitive to task-relevant semantic information also exhibit sensitivity when object meaning is task-irrelevant. The logic directly follows from Experiment 1: if spatial attention is biased toward the SR object, the object should receive a benefit from the increased attentional allocation, leading to a stronger neural representation. Thus, when two related objects are presented simultaneously, the added attentional benefit should support and ultimately enhance the representation of both objects. Comparatively, when the two objects are not related, the object representations should be weaker. This design is a follow-up to Experiment 1 in which this type of analysis was impossible, given that each object triad always contained one SR and one NR object. Additionally, unlike the task in Experiment 1, where effect was focused toward accuracy, the task in Experiment 2 pushed the effect into RTs.

In Experiment 2, participants were presented with two objects on either side of fixation that were either SR or NR (Fig. 2*B*) and performed a fixation task. We predicted a behavioral benefit when both objects are SR than when not and hypothesized that the increased attentional allocation from task-irrelevant semantic information will facilitate the strength of an object’s neural representation, leading to significantly higher classification accuracy of the object identity. Internally replicating previous behavioral findings, participants were significantly faster at performing the task when both objects were SR than NR (*t*(12) = 2.51, *P* = 0.028, Cohen’s *d* = 0.70; Fig. 2*E*) suggesting that attentional facilitation is directed by task-irrelevant semantic relationship between objects. Accuracy data between the SR (M = 96.8%) and NR (M = 95.6%) conditions also showed similar patterns as Experiment 1 but did not reach significance, *t*(12) = 1.65, *P* = 0.12. These behavioral results replicate our previous results and demonstrate the robustness of the influence of task-irrelevant high-level semantic relationship between objects on attentional allocation.

### Semantic Information Modulates Object Representations in the EVC

**Figure 4:**
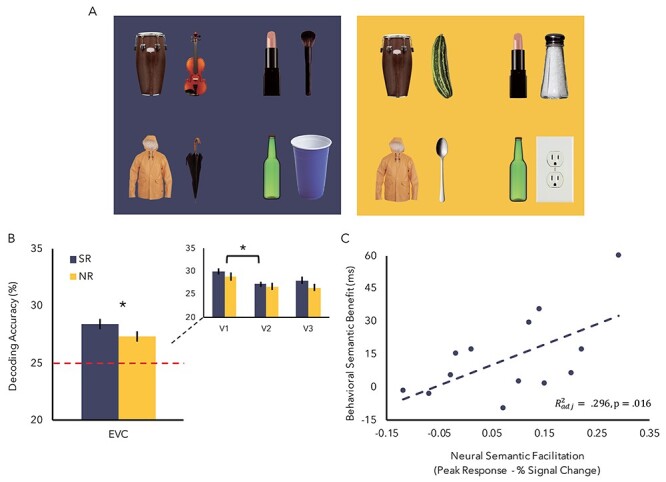
Experiment 2 stimuli and fMRI results. (*A*) Full example of the four main objects (left of each pair) when paired with an SR object or NR object. The object pairs in the blue background indicates SR condition, and the object pairs in the yellow background indicates NR condition. (*B*) Decoding accuracy performance of a linear classifier. Performance was only greater than chance (25%) in EVC. A main effect of semantic relatedness was also observed in EVC, with SR pairs having higher decoding accuracy than NR pairs. Small insert graph represents EVC data across V1, V2, and V3, showing a main effect of ROIs, such that V1 decoding accuracy was greatest. (*C*) Semantic facilitation in left IFG predicted behavioral benefit (^*^P < 0.05, and error bars represent ±1SE).

Using MVPA, we reasoned that if semantic information increases attentional allocation to an object, the strength of the object’s neural representation should be enhanced when paired with an SR object than an NR object. To test this, a linear SVM classifier was trained to discriminate between the four reference objects used in the experiment (Fig. 4*A*) with the prediction that decoding accuracy would be greater when a reference object was paired with an SR object than with an NR object, reflecting a less noisy representation supported by semantic influence from the frontal cortex. Classification performance of the SVM was tested against chance performance of 25% within the EVC (Fig. 4*B*). A three-way repeated measures ANOVA was conducted with hemisphere (left, right), ROI (V1, V2, V3), and semantic relationships (SR, NR) as within-subjects variables. There was a main effect of ROI (*F*(1, 24) = 5.27, *P* = 0.013, *η*^2^*_p_* = 0.305), with post hoc tests showing significantly higher decoding accuracy for V1 compared with V2, *t*(12) = 3.62, *P* = 0.01 (Bonferroni corrected). When compared against chance, both the SR (*t*(12) = 5.65, *P* < 0.001) and the NR condition (*t*(12) = 2.94, *P* = 0.01) were significantly greater than chance. Critically, a significant main effect of semantic relationships was observed, with decoding accuracy significantly higher for the SR than NR condition (*F*(1,12) = 5.69, *P* = 0.034, *η*^2^*_p_* = 0.332), suggesting that the benefit conveyed by an object’s presence to the neural representation of another object is greater when the objects are SR to one another. No other main effect or interaction reached significance (*F*s < 1). Consistent with the previous findings, our results demonstrate that the increased attentional allocation leads to a robust effect of task-irrelevant semantic information on the neural pattern of the objects represented in the EVC.

### Overall Semantic Facilitation in Left IFG Predicts Behavioral Benefit

Thus far, we provide evidence that task-irrelevant semantic information influences the efficiency of sensory processes in EVC through altering spatial priority maps in IPS. The consequence of which is enhanced representation of the objects in EVC. We next investigated how this high-level semantic information is represented in the brain. The left IFG has been implicated as central to the processing of task-relevant semantic information (Thompson-Schill et al. 1997; Wagner et al. 2001; Binder et al. 2009; Lupyan et al. 2010), with it playing a pivotal role in the selection of semantic knowledge. Considering the speed at which semantic information is processed (Potter and Levy 1969; Potter 1976; Thorpe et al. 1996; Potter et al. 2014) and its ubiquitous nature (Shomstein et al. 2019), we hypothesized that semantic information should be represented in the brain regardless of its task relevance. Thus, if attention is enhanced toward the SR object, the left IFG mechanism will be more engaged in the presence of an SR object than NR object.

Given two types of trials, one in which the object pair was either SR or NR, Experiment 2 allowed for a direct SR versus NR whole brain contrast to identify regions in the brain selective toward semantic information. For all participants, a whole brain univariate SR versus NR contrast was conducted, revealing the left IFG, which was then individually defined with a threshold of *P* < 0.05. For each individual, the strength of semantic facilitation was calculated as the difference between BOLD responses of SR and NR trials in the defined left IFG, with a more positive value representing stronger facilitation. To determine whether neural activity predicts behavior, the strength of semantic facilitation was again correlated with the difference in behavioral semantic facilitation (RT; NR—SR) revealing a significant positive correlation (*r* = 0.595, *P* = 0.032; Fig. 4*C*). This result demonstrates that the strength of neural semantic facilitation is directly connected to the strength of behavioral semantic influence.

## Discussion

All real-world objects in our environment are complex entities, described by basic physical properties and high-level semantic meaning. There has been an increasing interest to understand the role of semantic information in attentional guidance. Accordingly, it has been identified that semantic information is rapidly extracted and processed by a broadly distributed network (Binder et al. 2009) and that semantic relationships influence attentional allocation (Moores et al. 2003; Malcolm et al. 2016). However, the two types of findings remained largely independent, and thus the mechanism of semantic influence on attentional distribution remained ill-defined. Three key findings for the current investigation demonstrate that we have, for the first time, provided a potential mechanistic explanation of semantic influence on attentional allocation.

First, we provide evidence that even task-irrelevant semantic information impacts visual processing. Target discrimination performance was significantly more accurate for targets embedded within an object that was SR to the reference object than targets within a nonrelated object. This effect was internally replicated using a different paradigm in which participants were tasked to discriminate the orientation of Gabor patches embedded within a pair of SR or nonrelated objects. Performance was modulated by semantic relatedness, such that task performance was significantly faster when the objects were SR, demonstrating an increased efficiency in processing of visual information. Altogether, these results directly replicate findings that semantic relationships of task-irrelevant objects guide attention (Malcolm et al. 2016; Cornelissen and Võ 2017; Peacock et al. 2018) and provide strong evidence that objects’ semantic properties continuously influence visual attention allocation even when the information is not task-relevant. Importantly, these findings demonstrate that semantic information is processed obligatory and continually influences attention.

Second, this behavioral modulation is accompanied by changes in the overall response and strength of object representations in early visual cortices. BOLD activity in early visual sensory areas (V1–V3) was modulated by task-irrelevant semantic information, demonstrating that associative knowledge between objects modulates neural activity in the EVC. Additionally, using multivariate measures, we revealed that an object’s neural representation was enhanced when supported by a semantically related object. These results provide the first demonstration that the semantic context of an object (i.e., its relationship to other nonretinotopically coincident objects) affects its representation at the earliest stages of visual processing.

Finally, we show that the same regions known to mediate spatial attentional shifts (IPS) (Corbetta and Shulman 2002; Serences and Yantis 2007; Silver and Kastner 2009; Shomstein 2012) and process semantic information (left IFG) (Thompson-Schill et al. 1997; Wagner et al. 2001; Binder et al. 2009) are modulated by semantic relationships. In the case of left IFG, these modulations strongly predict the strength of semantic behavioral facilitation. BOLD response in spatially selective IPS was modulated by task-irrelevant semantic information, suggesting that the modulation of sensory representations of objects in the EVC is accomplished through facilitation of spatial representations. While left IFG has been demonstrated to be involved in processing of semantic information (Gabrieli et al. 1996; Fiez 1997; Thompson-Schill et al. 1997; Wagner et al. 1997; Wagner et al. 2001), the current study demonstrates that its involvement is not restricted to task-relevant situations. Thus, the results offer evidence that high-level semantic influence on attentional allocation is achieved through the processing of semantic relationships in the left IFG, which modulates spatial priority maps in IPS, and ultimately influences early sensory representations. The task-irrelevant aspect of this modulation shows that this process is not susceptible to task demands, and rather is ongoing and automatic.

A possible alternative explanation of the semantic benefit can be found in the configuration of the objects. SR objects were arranged in a manner often encountered in the real-world (e.g., raincoat is more likely to be found next to an umbrella than a spoon). Studies have provided evidence that object configuration influences object perception and attention (Green and Hummel 2006; Roberts and Humphreys 2011) as well as decrease neural interference (Kaiser et al. 2014). However, semantic similarity can be defined as the repeated co-exposure of visual objects that are physically proximal and are meaningfully related within a specific scenario or event (in fact, the concept literature defines this as thematic similarity; see Mirman et al. 2017 for review). The semantic effect observed in the current study is investigating the influence objects that functionally belong with each other and also co-occur in the real-world have on attentional allocation. Thus, frequently co-occurring objects in the real-world (e.g., lipstick and make-up brush, a violin and drum) are considered to be SR and is a defining feature of the two objects’ relationship.

Our findings provide both behavioral and neural evidence uncovering a link between task-irrelevant conceptual information and the perceptual system (Greene and Fei-Fei 2014). While recent neuroimaging studies have provided evidence that semantic and visual information can coexist within the same brain region (Walther et al. 2009), these studies were mainly restricted to task-relevant semantic information, thus offering no insight to the important question of whether semantic influence on perception is automatic. There has also been a recent effort to understand how semantic information affects attention and perception even in task-irrelevant situations (Greene and Fei-Fei 2014; Malcolm et al. 2016; Cornelissen and Võ 2017), given the ubiquitous nature of semantic information. In summary, we identify, and describe, a potential neural network through which high-level information increases efficiency within sensory processing regions in the brain. Task-irrelevant semantic information is processed in the left IFG, altering the priority weightings in the spatial attention maps within the parietal cortex, which in turn modulate activity in the EVC. Thus, these results provide converging behavioral and neuroimaging evidence that the impact of task-irrelevant semantic information extends to the lowest and highest levels of visual processing.

## Supplementary Material

nah-et-al_semTriads-SOM_ccc_tgab049

## Notes

J.C.N., G.L.M., and S.S. all contributed to theoretical motivation, developed study design, and wrote the paper. J.C.N. and G.L.M. programmed the experiment. J.C.N. conducted the experiments, performed data collection and analysis. The funders had no influence in study design, data collection and analysis, preparation of the manuscript, or the decision to publish. We thank Dr Dwight Kravitz for insightful comments on an earlier version of the manuscript.
