# Supplementary material for: Task-Irrelevant Semantic Properties of Objects Impinge on Sensory Representations within the Early Visual Cortex
Source: Cereb Cortex Commun. 2021 Aug 10;2(3):tgab049. doi: 10.1093/texcom/tgab049 (PMC8382923; doi:10.1093/texcom/tgab049)
Supplement: nah-et-al_semTriads-SOM_ccc_tgab049 [file nah-et-al_semtriads-som_ccc_tgab049.docx]

**Table 1.** Region of interests (ROI) voxel size and Talairach coordinates for Experiment 1

| ROI | Hemisphere | TAL Coordinates | | | Voxels |
| --- | --- | --- | --- | --- | --- |
|  |  | x | y | z | (1 x 1 x 1 mm) |
| V1 | Left | -8 | -94 | -4 | 1614 |
| V2 |  | -12 | -97 | 4 | 1687 |
| V3 |  | -19 | -95 | 6 | 1630 |
| LOC |  | -39 | -80 | -5 | 1277 |
| IPS0 |  | -25 | -80 | 22 | 2969 |
| IPS1 |  | -24 | -73 | 32 | 1817 |
| IPS2 |  | -21 | -68 | 39 | 1545 |
| V1 | Right | 12 | -90 | 3 | 1180 |
| V2 |  | 13 | -91 | 10 | 1320 |
| V3 |  | 18 | -90 | 11 | 1505 |
| LOC |  | 36 | -79 | -1 | 1281 |
| IPS0 |  | 24 | -77 | 26 | 2769 |
| IPS1 |  | 24 | -69 | 36 | 1885 |
| IPS2 |  | 22 | -66 | 44 | 1967 |

**Table 2.** Region of interests (ROI) voxel size and Talairach coordinates for Experiment 2

| ROI | Hemisphere | TAL Coordinates | | | Voxels |
| --- | --- | --- | --- | --- | --- |
|  |  | x | y | z | (1 x 1 x 1 mm) |
| V1 | Left | -9 | -90 | -8 | 8805 |
| V2 |  | -13 | -86 | -5 | 6813 |
| V3 |  | -21 | -82 | -6 | 5299 |
| V1 | Right | 9 | -87 | -5 | 7865 |
| V2 |  | 11 | -84 | -3 | 6491 |
| V3 |  | 18 | -81 | -4 | 5274 |
